# Giant cell tumor of the hand in a pediatric patient: A case report

**DOI:** 10.1016/j.ijscr.2025.111763

**Published:** 2025-08-05

**Authors:** Herry Herman, Adrian Fakhri Ismiarto, Hans Kristian Handoko, Yosep A. Tarong, Sebastian Chendra

**Affiliations:** a Department of Orthopaedics and Traumatology, Faculty of Medicine Universitas Padjadjaran, Bandung, West Java, Indonesia

**Keywords:** Giant cell tumor, Curettage, Bone graft, Local excision, Case report

## Abstract

**Introduction and importance:**

Giant cell tumors (GCTs) of the hand are rare and typically present as painful, locally expanding masses. Bone grafting is commonly used after tumor removal to fill defects and restore structural integrity. This case highlights the management of a suspected GCT in a child using curettage, bone grafting, and biopsy.

**Presentation of case:**

A 12-year-old girl presented with a progressively enlarging lump on her right hand. Examination revealed a solid, immobile mass (3 × 2 × 1.5 cm) with a well-defined margin and reduced distal neurovascular function. The capillary refill time was less than 2 s. Surgical management included curettage, iliac bone graft harvesting, graft insertion, and fixation with a K-wire.

**Clinical discussion:**

Curettage with bone grafting is the preferred treatment for GCTs, but it is associated with a risk of recurrence, especially in pediatric patients, due to rapid bone turnover and anatomical challenges in the hand. Iliac bone grafts provide structural support after curettage. Follow-up imaging is essential to detect early recurrence.

**Conclusions:**

Wide-scale local excision may offer better local control but can result in morbidity, particularly in the hands. Further research is required to clarify the long-term outcomes and optimize adjuvant therapy strategies.

## Highlights

• Rare hand GCT in a 12-year-old girl highlighting diagnostic and management challenges is described in this case report.
• Treatment included curretage, iliac bone graft, and K-wire to preserve hand function.
• Pediatric GCTs are rare, slightly more common in females, possibly due to puberty-related hormones.
• GCTs have up to 45% recurrence, requiring regular follow up and early imaging.
• Surgery includes curretage with adjuvants or wide excision, the latter has less recurrence but higher morbidity

## 1 Introduction

Clinically, giant cell tumors (GCTs) of the hand typically present as a painful and locally expanding mass. The symptoms include swelling, tenderness, limited range of motion, and possible pathological fractures. Radiographically, these tumors appear as a well-defined, eccentrically located, lytic lesion with distinct margins. Magnetic resonance imaging (MRI) is often used to assess the extent of the tumor and to evaluate its relationship with adjacent structures [1,2].

The treatment of GCTs of the hand usually involves a combination of surgical intervention and adjuvant therapies. The primary goal of this approach is to achieve complete tumor removal while preserving hand function. Curettage, where the tumor is excised, and bone grafting are commonly performed. In more extensive cases or cases with recurrence, en bloc resection, which involves removing the affected bone segment, may be necessary [1]. Bone grafting is commonly used as an adjuvant procedure following tumor removal to fill the void created by the excised tumor and promote bone healing and structural stability. It plays a crucial role in restoring function, preventing deformity, and reducing the risk of recurrence [1,2].

The choice of bone graft material depends on various factors, including the size and location of the bone defect, patient factors (such as age and comorbidities), surgeon preference, and availability of graft options. During the surgical procedure, after the tumor removal, the bone defect is meticulously prepared and shaped to accommodate the bone graft. The graft is then inserted into the defect, ensuring good contact between the graft and the remaining healthy bone. Fixation techniques, such as plates, screws, or wires, can be used to stabilize the graft and promote healing [3,4]. In some cases, bone grafts may be combined with other adjuvant treatments, such as bone cement or bone substitutes, to enhance graft stability and bone healing. The aim of this review is to assess the role of bone grafting in the management of GCT of the hand, including curettage, bone graft insertion, and biopsy. This article has been reported in line with the SCARE criteria [5].

## 2 Case presentation

A 12-year-old right-handed girl was referred to a tertiary hospital with a progressively enlarging lump on her right hand. The mass had been first noticed approximately one month prior to admission, initially the size of a marble. Over time, the lump had increased in size and was now associated with intermittent numbness, particularly during manual activities. Despite the progressive nature of the swelling, the patient remained systemically well. She denied experiencing any constitutional symptoms such as fever, fatigue, loss of appetite, weight loss, or night sweats. There was no history of similar lumps in other parts of the body.

The patient had initially presented to a clinic, where a preliminary evaluation was undertaken. Given the clinical concern, she was subsequently referred to the tertiary care facility for further assessment and management. Her past medical history was unremarkable, with no previous hospitalizations, surgical procedures, or chronic illnesses reported. She was not taking any regular medications at the time of presentation, and there was no history of drug allergies or adverse drug reactions. No contraindications to initiating or resuming medical therapy were identified.

The patient lived at home with her family and was socially dependent, which was appropriate for her age. She denied any history of tobacco use, alcohol consumption, or illicit drug use. A detailed family history was obtained, which revealed no known hereditary disorders, autoimmune diseases, familial malignancies, or similar skeletal or bone-related conditions in first-degree relatives. This reduced the likelihood of systemic skeletal diseases or underlying genetic syndromes. A general review of systems was unremarkable. The patient did not report any headaches, visual disturbances, palpitations, chest pain, gastrointestinal symptoms, or joint discomfort. Her presentation was therefore focused on the localized mass and its neurological impact on hand function.

**Fig. 1:**
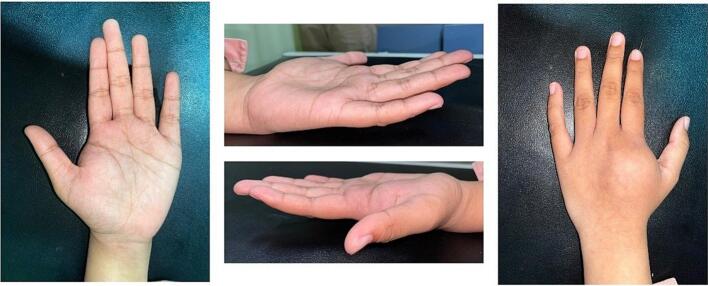
Lump at 2nd metacarpal left hand.

**Fig. 2:**
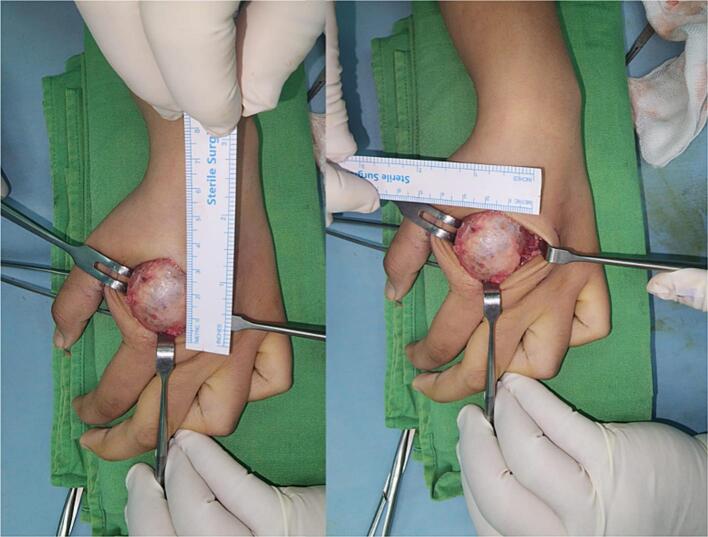
Solid lump measuring 3 × 2 × 1.5 cm.

Physical examination revealed the presence of a lump (Fig. 1). However, other associated findings, such as venous distention, distal swelling, *peau d'orange* (a dimpled appearance of the skin resembling an orange peel), and fungating wound were not observed. However, a comprehensive examination of the upper extremities revealed the presence of a solid lump measuring 3 × 2 × 1.5 cm (Fig. 2). The lump exhibited a regular surface, well-defined margin, immobile, and reduced distal neurovascular function compared with the unaffected side. The capillary refill time was less than 2 s, indicating good vascular perfusion. However, the range of motion of the metacarpal joint was limited due to the presence of lumps. We performed pre-operative X-ray but advanced imaging studies such as CT or MRI of the hand were not performed due to resource limitations at the time of evaluation. The lesion is most consistent with a Campanacci Grade III giant cell tumor.

Preoperative functional evaluation was conducted using the Pediatric Outcomes Data Collection Instrument (PODCI). The results indicated only mild impairment in upper extremity function, with preserved fine motor skills and the ability to perform daily activities independently. Given the diagnosis of a Campanacci Grade III giant cell tumor, metastatic assessment particularly to the lungs, is clinically warranted, as pulmonary metastases, although rare, have been reported in up to 6 % of cases. In this patient, a chest X-ray was performed with normal result, but due to financial constraints, advanced imaging such as PET scan or whole-body scan was not performed.

**Fig. 3:**
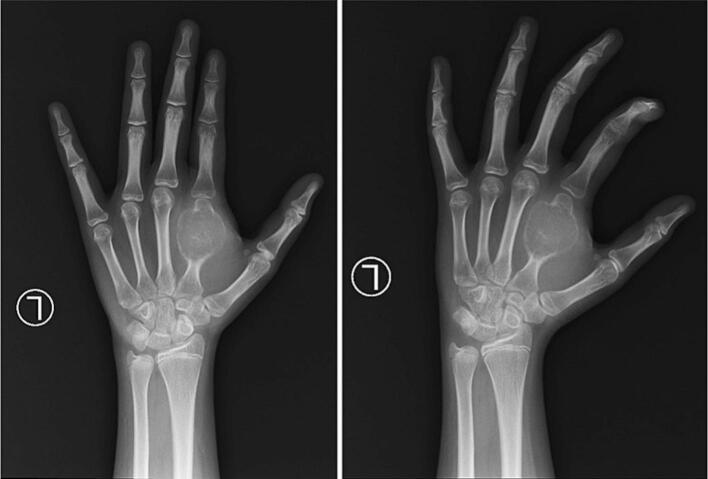
Bone tumor affecting the 2nd metacarpal.

**Fig. 4:**
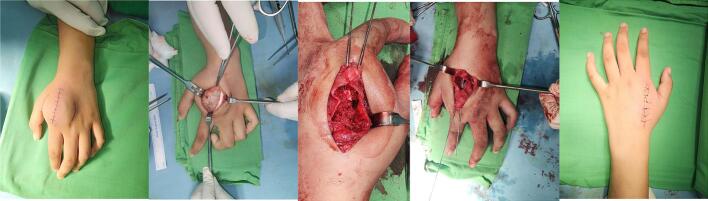
The surgical procedure involved curettage (removal of the tumor tissue by scraping or scooping), harvesting of the bone graft from the iliac bone, insertion of the graft, and internal fixation using a K-wire.

Based on these clinical findings, a provisional diagnosis of suspected GCT was established as the primary bone tumor affecting the 2nd metacarpal (Fig. 3). A surgical intervention was considered appropriate for managing this condition. The surgical procedure involved curettage (removal of the tumor tissue by scraping or scooping), harvesting of the bone graft from the iliac bone, insertion of the graft, and internal fixation using a K-wire (Fig. 4). The surgery was performed by a senior surgeon. The decision to use an autologous pelvic bone graft rather than a commercially available synthetic bone substitute (e.g., bio-bone or allograft) was made primarily due to financial limitations, as the patient and her family were unable to afford the cost of synthetic graft materials. While we acknowledge that harvesting bone from the pelvic region can raise aesthetic and donor site morbidity concerns, especially in young female patients, autologous grafting remains a viable and effective option in resource-limited settings. Efforts were made to minimize the incision and donor site complications through careful surgical technique.

**Fig. 5:**
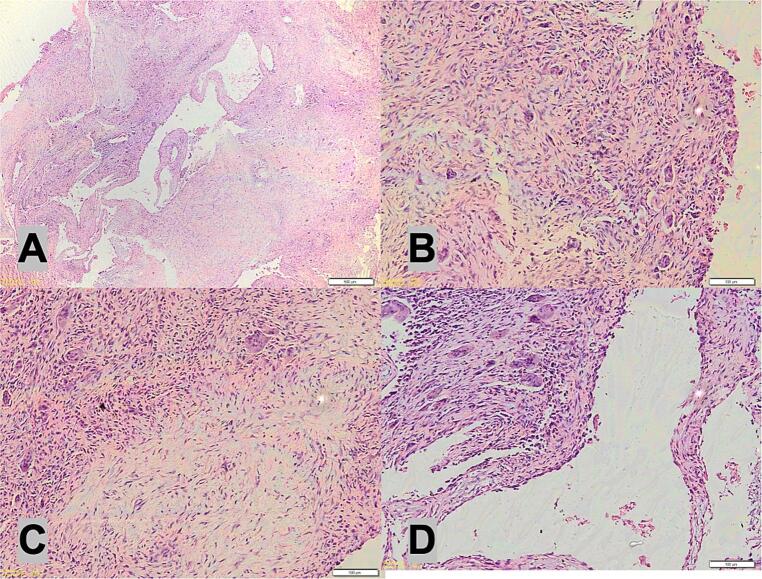
The histology examination show multinucleated giant cells.

**Fig. 6:**
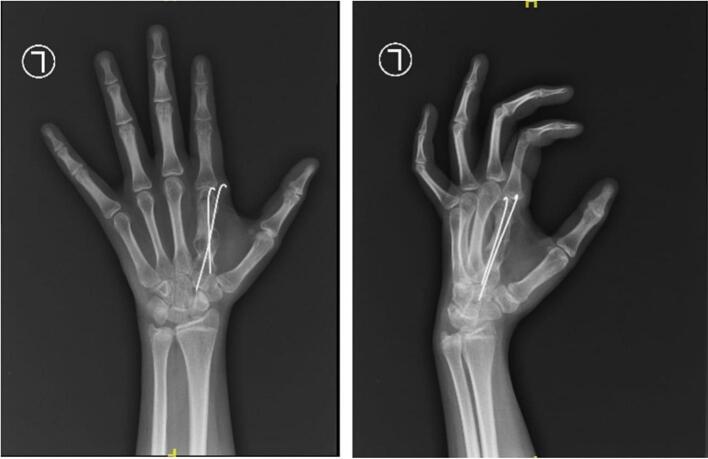
3-month follow-up, we conducted evaluation with X-ray, and there were no signs of recurrent GCT.

**Fig. 7:**
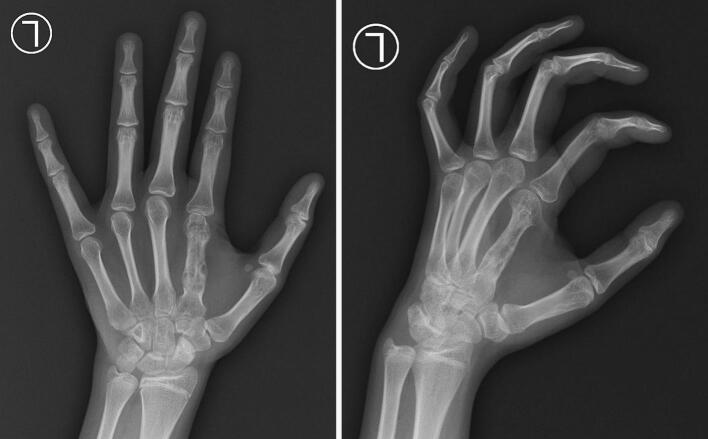
1-year follow-up, we conducted evaluation with X-ray, and there were no signs of recurrent GCT.

During surgery, after tumor excision and curettage, the surgeon performed thorough mechanical debridement of the cavity wall to minimize residual tumor cells. We performed an immediate frozen section biopsy. The histology examination is depicted in Fig. 5. At the 3-month follow-up and 1-year follow-up, we conducted evaluation with X-ray, and there were no signs of recurrent GCT (Fig. 6 and Fig. 7). The PODCI evaluation was repeated to evaluate functional recovery. The results demonstrated improvement in upper extremity function and pain scores, with the patient reporting increased ease in performing daily activities and better hand mobility. No major complications or new limitations were reported at the donor or operative site during follow-up. Denosumab was not administered due to the localized nature of the tumor, the feasibility of surgical resection, and financial limitations. Post-operative radiotherapy is not performed due to the risk of radiation-induced sarcoma, especially in younger patients. It is typically reserved for recurrent, residual, or inoperable tumors or where surgical margins are uncertain.

## 3 Discussion

The hand plays an important role in function. This case highlights the importance of thorough clinical examination and medical history assessment when evaluating patients with unusual lumps or growths. The progression of the lump in size and the presence of associated symptoms, such as occasional numbness, can provide valuable diagnostic clues. Referral to a specialized medical centers is helpful for the further evaluation and management of complex cases. The absence of typical signs, such as venous distention and peau d'orange, underscores the need for a comprehensive examination to identify the specific characteristics of the lump. Surgical intervention, including curettage, bone grafting, and internal fixation, can be an effective treatment approach for suspected bone tumors in appropriate cases.

GCT usually occur in young adults aged 20–40 years [6]. The occurrence of GCT in the hand in pediatric patients is rare, and the incidence varies from 1.8 % to 10.6 % [7]. This tumor has a slight female predominance [[7], [8], [9], [10]]. However, another study also reported male dominance [7]. The reason for the slight predominance of GCT in females is not fully understood, but several factors may contribute to this trend. A key consideration is the hormonal changes occurring during puberty. In females, a surge in estrogen and other reproductive hormones during adolescence may play a role in bone metabolism and cellular activity, which could contribute to the development of GCT. Estrogen may influence osteoclast function, possibly contributing to the abnormal growth patterns observed in GCT. Additionally, skeletal development during puberty, especially in areas with rapid growth and high bone turnover, might make bones more susceptible to tumor formation. Although the presence of hormone receptors in GCT cells has been suggested, further research is needed to fully understand the molecular mechanisms underlying female predominance in pediatric cases [6,11,12].

In pediatric cases, GCT commonly present with asymptomatic lumps [[7], [8], [9]]. However, symptoms such as pain, swelling, and limited range of motion can occur, often due to the tumor's local expansion and interaction with surrounding bone and soft tissues. Pain is typically the first symptom noticed, as the tumor's growth exerts pressure on the bone and nerves, leading to discomfort that can worsen with activity or at night. Swelling is another hallmark feature, as the tumor creates a visible mass or lump, often accompanied by tenderness to touch. The affected area may appear enlarged due to the tumor's invasion of the bone and nearby tissues. Limited range of motion occurs when the tumor is located near a joint, such as in the hand, thereby restricting normal movement. This condition can interfere with daily activities, particularly in children, as joint stiffness and discomfort can severely impact the ability of the affected limb to function. In severe cases, if the tumor is not addressed in time, it can lead to functional impairment or even pathological fractures, where the bone becomes weakened and susceptible to breaking with minimal trauma. These symptoms, while common, are nonspecific and may be mistaken for other benign conditions, underscoring the need for prompt evaluation in pediatric patients [8].

The current local treatment options for GCT of the bone include intralesional curettage with adjuvant therapy and wide local excision [4,[13], [14], [15]]. Intralesional curettage involves removing the tumor by scraping or scooping it out, followed by adjuvant therapy such as phenol, liquid nitrogen, or bone cement to destroy any remaining tumor cells and reduce the risk of recurrence. Wide local excision involves removing the tumor along with a margin of healthy tissue to ensure complete tumor removal. However, wide local excision can result in significant morbidity, especially in cases where major joint reconstruction is required. Therefore, the choice of treatment depends on several factors, such as the grade and location of the tumor, as well as the patient's overall health and functional goals [14]. A previous study reported that wide resection is associated with a lower recurrence rate compared to intralesional surgery, with rates of 5 % versus 25 %, respectively. The use of polymethylmethacrylate (PMMA) following intralesional surgery reduces the likelihood of local recurrence compared to bone grafting, whereas phenol application alone does not influence recurrence risk. These surgical options are considered the mainstay of treatment for GCT because they offer the best chance of cure and local control. Other treatment modalities, such as radiation therapy and systemic therapies, may be used in certain cases where surgery is not feasible or as adjuvant treatments. However, surgery remains the primary approach.

Previous studies have reported no recurrence for up to 1 and 3 years after complete excision [8,9]. The largest case series of GCT in pediatrics reported that the preferred treatment is complete surgical excision. Even though the study was conducted nearly two decades ago, almost all of the surgeries were without complications, and all parents were satisfied with the results. The common complications were superficial wound infection and scar tissue [10]. Several unanswered questions remain regarding the use of denosumab in the management of GCT. These include the optimal treatment duration for unresectable diseases or cases in which treatment is given in the neoadjuvant setting. The feasibility of interval therapy and the morbidity and risk of long-term therapy should also be considered. Another important question is the risk of recurrence after discontinuing denosumab. Studies have shown that the risk of recurrence increases after discontinuing denosumab, with a median time of recurrence of approximately 8 months. It is unclear whether there is a benefit to adding denosumab in the adjuvant setting to reduce the risk of recurrence.

GCT are known for their high recurrence rate, which has been reported to be up to 45 % [[8], [9], [10]]. The tendency for recurrence is often attributed to the biological behavior of the tumor, which can infiltrate surrounding bone and soft tissues, making complete removal difficult. Incomplete resection is one of the primary risk factors for recurrence. If even a small portion of the tumor remains after surgery, the residual tumor cells can proliferate and cause regrowth. This risk is particularly significant in pediatric cases where surgeons may attempt to balance tumor removal with preservation of bone and joint function, especially in the hand, which is a highly functional and delicate region. Patient age is another critical factor. Younger patients tend to have a higher recurrence rate, possibly due to the higher rate of bone turnover and growth, which may contribute to a more aggressive tumor behavior. Tumor biology in pediatric patients may also differ from that in adults, with younger individuals potentially experiencing more active cell proliferation, thus increasing the risk of tumor regression. Tumor size also influences the risk of recurrence. Larger tumors, particularly those that have extensively invaded bone or joint structures, are more difficult to completely excise, increasing the likelihood of recurrence. Additionally, larger tumors may exhibit more aggressive biological behavior, further contributing to this risk.

Given these recurrence risks, regular follow-up is crucial for the early detection and management of any signs of recurrence. Monitoring typically includes periodic imaging, such as X-rays or MRI, to evaluate the surgical site and surrounding tissues. Early detection of recurrent GCT allows for timely intervention, potentially involving additional surgery or adjuvant treatments like cryotherapy, phenol application, or systemic therapies such as denosumab. Without regular follow-up, recurrent tumors may progress and cause significant complications, including bone destruction, functional impairment, and the need for more extensive surgical interventions. Therefore, consistent monitoring and early detection are essential for improving long-term outcomes among patients with GCT.

Timely recognition and thorough investigation are essential when progressive growth of a lump in the hand is present, particularly in the presence of occasional numbness. A comprehensive physical examination, including evaluation of size, surface characteristics, mobility, and neurovascular function, plays a critical role in assessing suspected bone tumors. Referral to a specialized hospital proficient in managing bone tumors is crucial for accurate diagnosis and appropriate management. However, failure to consider the possibility of a bone tumor in a young patient with a growing hand lump may result in delayed diagnosis and treatment. Relying solely on the absence of specific associated findings, like venous distention and distal swelling, may lead to overlooking significant clinical features. Furthermore, a limited joint range of motion due to a tumor should raise suspicion of the underlying pathology and prompt further investigation.

One of the strengths of this case lies in its clear and progressive clinical presentation, which highlights a common yet diagnostically diverse symptom—an enlarging hand mass in a pediatric patient. The case underscores the importance of timely referral from a primary facility to a tertiary center, enabling comprehensive diagnostic evaluation and multidisciplinary management. Additionally, the absence of systemic symptoms and a well-documented clinical course provides a focused context for diagnostic reasoning. However, a limitation of this case is the lack of histopathological confirmation or imaging findings at the time of reporting, which restricts the ability to draw definitive conclusions about the etiology of the mass. Furthermore, the absence of long-term follow-up data limits the discussion on treatment outcomes and prognosis.

## 4 Conclusions

GCT of the hand is a rare but challenging condition that requires prompt diagnosis and comprehensive management to preserve hand function and minimize the risk of recurrence. In this case, surgical intervention involving curettage, bone grafting, and internal fixation proved to be an effective treatment strategy. Although wide local excision offers the greatest chance for complete tumor removal, it must be carefully weighed against the potential complications, including surgical morbidity and long-term functional impairment. Ongoing follow-up is crucial for the early detection of recurrence, and further research is necessary to determine the optimal treatment duration and the role of adjuvant therapies in improving outcomes for patients with GCT of the hand.

## Consent

The patient's parents/legal guardian received an explanation of the procedures and possible risks of the surgery and gave written informed consent. My manuscript does not contain any individual person data. Written informed consent was obtained from the patient's parents/legal guardian for publication and any accompanying images. A copy of the written consent is available for review by the Editor-in-Chief of this journal on request.

## Ethical approval

Ethical approval for this study was provided by the Ethical Committee of Medical Faculty, XXX University on March 20, 2025.

## Guarantor

The guarantor in this study is Hans Kristian Handoko.

## Funding

None.

## Author contribution

Herry Herman:Surgeon, Conceptualization, Visualization, Methodology,

Writing and Supervision.

Adrian Fakhri Ismiarto: Surgeon, supervision and writing

Hans Kristian Handoko: Writing

Yoseph A Tarong: Writing

Sebastian Chendra: Writing

## Declaration of competing interest

All authors have completed the ICMJE uniform disclosure form. The authors declare no conflicts of interest.
